# Supplementary material for: Genomics analysis of genes encoding respiratory burst oxidase homologs (RBOHs) in jatropha and the comparison with castor bean
Source: PeerJ. 2019 Jul 11;7:e7263. doi: 10.7717/peerj.7263 (PMC6626655; doi:10.7717/peerj.7263)
Supplement: File S1 [file peerj-07-7263-s001.pdf]

Supplementary File S1. The gene model for *JcRbohN*. The coding region is marked with uppercase letters, above which is its deduced amino acids. The transcribed untranslated regions, including 5’ UTR, intron and 3’ UTR sequences, are marked with lowercase letters. The start and stop codons are marked with bold letters.

```
   1 ccatttcccacactcaaccactaaaatcccagaaaaacattaatctcttctccttaacct
  61 acatgcatgcatttctccacagataacccctaaacatttttttttttgttttctctacca
 121 agaattctagtccattaatggagtctcaagtagagtcatctctttctttttcttcttctt
 181 cttcttcttcttcttcttttttcttcttcttcttcatcatggagtggtagagttcctc
   1                                            M  E  D  E  E  T
 241 ctctttcagtctctcaaggaacagaatttgaccagcaccatgtgATGGAGGATGAGGAGA
   7  S  V  A  L  G  E  R  L  R  F  I  D  I  E  W  K  E  V  E  K
 301 CATCAGTGGCACTTGGTGAACGCCTGAGATTCATTGACATTGAATGGAAGGAAGTGGAAA
  27  R  F  D  R  L  A  F  T  G  K  A  P  E  P  V  V  K  W  S  D
 361 AACGTTTTGATAGGCTGGCTTTCACCGGAAAAGCCCCTGAACCGGTCGTGAAGTGGTCGG
  47  F  G  F  C  I  G
 421 ATTTTGGTTTTTGTATAGgtaacaagaattgtctattaagtctcaaatattttgcttttc
 481 caattgagacaaaataagggtaacgcacaaattgcatgagattttgttcatggcatcact
 541 atcttctttccctttttgaccaaatcttttcttgaatatttctagaaaaaacataatgta
 601 ataattatttggtaaatatgattagggtaattatttgggatttcatcacaatttttgttg
 661 acttcccaaagaattttccaatttcaaaattgactttttactaatttctatattgttatg
 721 ttttaaaaaaactcataatttcagcatattttaatgttttcgattacaaatacaactgtt
 781 tttttcaaatattagagaggggggattcgaatctgaatttattactaggtcctatatgcac
 841 ttactactatgctatacattggggtgtcacaaatatatatatttttttatttctacattat
 901 tacacattttaaatccttttatttaataatttttttataaagtaatgcaattttaaatta
 961 tgtacttagctaagctttattcaagaaactgattcctatataataatagagtgtatgata
1021 gtcaaccatacaagaataataatagaagtctcaacaactttttattggtgtgctttttgg
1081 gcatccctcacttttttttttctttttacacaatattattaaaagattatcctaatatt
```

1141 ggttaaaaaatgaatctagccaaccactaaattatattcgaggttataataatttgcata

1201 gtggttaatttcttgtatcaatactaatcaaatatgatttattaattaatgttgacaaag

1261 gaaataaaaaccctttacctaaaatatttttactactactattttattttaatttatttt

1321 ttgttgttttgtaatgctcttttttttaatttaattactaaaaaaaatattgcctttttt

53 M Q Q S P E F A K E V L M A I R

1381 atttctacagGTATGCAACAATCACCAGAATTTGCTAAAGAGGTTTTAATGGCAATAAGA

69 G R G K K S K I D M T K R E L H G Y W H

1441 GGAAGAGGAAAAAAAAGTAAAATTGATATGACAAAAAGAGAACTCCATGGTTACTGGCAT

89 R L T D P C F N S R V K I F F D M

1501 AGATTGACTGACCCTTGCTTCAATTCAAGAGTGAAAATTTTCTTTGACATgtaatttatc

1561 aataatgattctgcaaattcttttttttttttttttttttgaaaagcaaattcttatttt

1621 tctttgacatggaattctttatatatatatatatatatatatatatatatataaatgtttat

106 C D

1681 ccaaccaatatttctgataggttctttttttcattcaatggcttttttttccttcagGTGTG

108 K N M D G R I N K M D I K Q

1741 ACAAAAATATGGATGGAAGAATTAATAAGATGGACATCAAGCAGgttgggtcaaatttcc

1801 ctatctacatatattatatattcaaattatacattggaggttgctcacctagtacaagaa

1861 aaccaaagttttaaacatgcttttttttttccttaattaattctgtttgaaaaaaaaaattc

1921 aattctttcatttgaataaggtaatttacagaacgcaaatacttttgaagtattataggt

1981 catataattggattgtttttttttttctagtctgtatattgagcgatggaaaatcgaatc

2041 taatttttctcaagttatatacgtacttattatcaagagtgctgtataattttaaattta

2101 tattttaatctaagaacaagcattgggtcaattttgttcgattttcacaaaattaaaata

122 N I

2161 atcgagaccaaaaaaatttcaaaaattattaaaattttgttttggacttttgacagAATA

124 L L I A S T N K L S L T Q D Q A E E N A

2221 TTTTGCTGATTGCTTCAACAAATAAATTGTCTCTAACACAAGACCAAGCAGAGGAAAATG

144 T L I M E A L D T Q G R G F I E

2281 CTACTTTAATCATGGAAGCTCTTGACACTCAAGGCCGGGGCTTTATCGAGgtgctcaatt

2341 taaactttttttattcatatatttataaggttttatcaattttaatctaattaataattaa

160 L S Q L A A L F K I S L P M R G S

2401 tttgcatgaagCTATCTCAACTAGCAGCTCTCTTCAAAATAAGCTTACCAATGAGGGGCT

177 L L P N R N H K S N S D L E D H Q D D Q

2461 CACTATTACCCAACAGAAATCACAAAAGTAACAGTGACCTGGAAGATCACCAAGATGATC

197 P I M S K P E V L F R A H W R R G W I V

2521 AACCAATCATGTCAAAGCCAGAAGTGTTGTTTCGAGCTCATTGGAGGAGAGGATGGATAG

217 V I W L V I C L S L F T W K F I Q Y K H

2581 TTGTGATTTGGTTGGTGATTTGCCTGTCACTTTTCACATGGAAGTTTATTCAATACAAGC

237 R T A F E V M G Y C L C T A K G A A E T

2641 ATAGAACAGCTTTTGAAGTAATGGGTTATTGCCTTTGCACTGCTAAAGGAGCAGCTGAGA

257 L K F N M A V I L L P V C R N T I T W L

2701 CCTTAAAATTTAACATGGCTGTCATTCTTCTTCCTGTTTGTAGAAATACCATTACATGGC

277 R R K A T I N S F V P F N D N I N F H K

2761 TTCGCAGGAAAGCAACAATCAATTCTTTTGTACCCTTCAACGATAATATCAACTTCCACA

297

2821 AGgtaatttttatttttatctttctatcaatatttgtttccgtacaatatatttcatttt

2881 cgttgtacagtctttctcttataagttcgaccttatacagaaccatcttgttaaaaaaat

2941 atcaacctttctattggagcatgtaaaatataatctaatcaaaaggtttgcaaccaataa

297 L I A G G I V V G V L L H G G T H L

3001 atacagTTAATTGCAGGAGGAATAGTGGTTGGTGTTCTACTCCATGGAGGAACTCATCTA

315 A C D F P R I S G S N R S I F R Q T I A

3061 GCTTGTGATTTTCCGAGAATTAGTGGATCTAATCGATCAATTTTCCGGCAAACTATTGCC

335 A R F G F H Q P S Y V Q I L T T T E V A

3121 GCCCGATTTGGGTTCCATCAACCTTCGTATGTACAAATATTAACCACGACAGAAGTAGCA

355 T G I A M V I L M A I A F L L A T K W P

3181 ACGGGAATTGCAATGGTAATACTAATGGCAATTGCATTTTTGCTAGCAACAAAATGGCCC

375 R R Q S P L L P K S V R N V T G Y N A F

3241 CGTCGCCAATCGCCTTTGCTGCCTAAATCTGTTAGAAATGTGACTGGTTATAACGCTTTC

395 W Y S H H L L I L V Y V L L I V H S M F

3301 TGGTACTCACATCACTTGCTCATTCTTGTCTACGTCCTGCTCATTGTTCATTCAATGTTC

415 L F L T D N V T E K T

3361 CTCTTTCTAACAGACAACGTAACTGAAAAAACGgtaagcatgcattttgttgaaactttc

3421 agaaattatctatttcgcctcctcaaaattcataaaattatatttgcttattttttgta

426 T W M Y V A F P I M L Y T G E R F V R A

3481 gACATGGATGTACGTTGCATTTCCAATCATGTTATACACCGGAGAGCGTTTTGTTCGAGC

446 I R S G F Y D A K I L K

3541 CATAAGATCAGGGTTTTACGATGCAAAGATTTTGAAGgtaactcactttcttaaaatgaa

3601 ataatcaagagcgcgtttaattcagctaattcatatcgaacaactagattatttaagtac

458 A S

3661 tataattattatattttttagctaattaagcactacgcttcactatatgagcagGCAAG

460 I Y P G K V L C L K L H K P E G F K Y K

3721 TATTTATCCAGGGAAAGTTTTGTGTCTCAAGTTGCACAAACCAGAAGGTTTCAAGTACAA

480 S G M Y I F L Q C P Q I S S F Q W

3781 GAGTGGGATGTATATTTTCTTACAATGCCCTCAAATTTCTTCATTTCAATGgtaactaaa

3841 ctacttataacaaacatttcaactagtaattaagtgatatttataatttaaattaactaa

497 H P F S L T

3901 tttcattatcttcttattgcttttgtcctttcccaatttcagGCACCCATTTTCATTAAC

503 S G P K D D H L S V H I R T L G D W S Y

3961 TTCAGGGCCAAAGGATGATCACCTAAGTGTCCATATTAGAACTCTTGGAGATTGGAGTTA

523 Q I Y S L F Q E

4021 TCAAATATACAGTCTCTTCCAAGAGgtaattatataaattcaccaatcaactttggttca

531 E I

4081 agattaatagaaagctaaatttataaaaagcttcaagattttatgagttttgcagGAAAT

533 L S G G V K T K Y P K I Y I D G P Y G S

4141 ATTATCTGGTGGAGTAAAGACGAAGTACCCAAAAATTTACATTGACGGACCTTATGGTTC

553 A S Q D Y V K Y D T V M L I G L G I G A

4201 TGCTTCACAAGATTATGTCAAGTATGATACAGTGATGTTAATTGGGCTTGGAATAGGAGC

573 T P F I S I L K D L A N F D Q

4261 CACACCTTTCATTAGCATCCTCAAAGATCTAGCTAATTTTGATCAGgtaattattctcca

4321 tctttcgaataaaactaattttgtcgaacgtgaattataataattgaaagagtatattct

4381 acttaaatttaaaaaaattagtcacaatctttcagttacttttaaaaatatagaaatata

4441 tatttatatagtcaataatttcagtcagaaatcatagtaatttatccaaatttctattac

588 A N C A

4501 ttaatggtgttatctaaccacataaaaaggtattctgactccatgcagGCCAATTGTGCA

592 E S N I S R G P L K A Y F Y W V T R E Q

4561 GAAAGCAACATTTCAAGAGGTCCATTAAAAGCTTATTTTTATTGGGTAACAAGAGAACAG

612 S S F A W F R D S L R E I S K T N E K L

4621 AGCTCCTTTGCTTGGTTTAGAGATAGCCTCAGGGAAATATCAAAAACAAATGAAAAACTG

4681 gtaattatttatatttaaacatttctttttctctctgttttttttttttttttttttct

632 A I I E M H N F

4741 aaccattaattcctaattttgtctttctccttttagGCTATTATTGAAATGCACAACTTC

640 L T S V Y G E G D A R S A L L S L I Q A

4801 TTAACTAGTGTATATGGAGAAGGAGATGCAAGATCTGCTTTATTAAGTCTCATTCAAGCA

660 L N Q T K N G V D V V S R T E

4861 TTGAACCAAACCAAAAATGGCGTTGATGTTGTATCTAGAACTGAGgtaactcatttaatt

4921 aaaattaaaataagatataatactatatttattattatttattaaaattgaaattgaaat

675 V P T Q F G R P N W F N I

4981 gtctaaccaatggagtttcttagGTACCAACACAATTTGGTCGTCCAAATTGGTTCAACA

688 F S K L A S R H R G E R I G

5041 TCTTCTCCAAATTGGCATCTAGACACAGAGGAGAACGTATTGgtaagctgattccccatt

5101 tctttctcaataatttagtactaattttaataacctgtaattaataattttaataacagt

5161 ttttaaataataatttaaggaagaaaattaggttggattgatttaatgattgttcttaga

5221 acacttaagaaataaagagaaaacaagatggacaagaggatttataatagtgtaagagag

5281 acaaatattaatttttttttcttaagaaaaattgagagtttgattgttatcaatgaagtaa

5341 atacccgttaagagtgctttattcttaataagctgataattacttattagggagtaaact

702 V F Y C

5401 caaatgataatacttaattaacaaacttttttttttctttggcatagGGGTGTTCTACTGT

706 G A S A L A R E L E R L C T N F S T K T

5461 GGCGCATCAGCACTAGCAAGGGAATTGGAGAGGTTGTGCACCAATTTCTCAACCAAAACA

726 T T R F V F H K E N Y *

5521 ACTACTAGATTTGTATTTCACAAGGAGAACTAT**TAG**acctcatacatagagcgatttaag

5581 aacatgaaattatttcattatttttcgagtaaatggaaaaaaattcatttatttttattt

5641 a
