# Supplementary material for: Genomics analysis of genes encoding respiratory burst oxidase homologs (RBOHs) in jatropha and the comparison with castor bean
Source: PeerJ. 2019 Jul 11;7:e7263. doi: 10.7717/peerj.7263 (PMC6626655; doi:10.7717/peerj.7263)
Supplement: File S2 [file peerj-07-7263-s002.pdf]

Supplementary File S2. The gene model for *RcRbohC*. The coding region is marked with uppercase letters, above which is its deduced amino acids. The transcribed untranslated regions, including 5' UTR, intron and 3' UTR sequences, are marked with lowercase letters. The start and stop codons are marked with bold letters.

1 ttttttctttctttacttcaatctgccacagttcaccttttaatctccgtcttcttgttc

61 tgagaatcataatctcgagtggtcagattcattattccatcttcaagattcaagcttttc

1 M Q K M G R E D

121 tgttttaacttccaagaattttgaatcaaaacccag**ATG**CAAAAGATGGGTAGAGAAGAT

9 N S H H Q H H H S D S E L I E G D Q R V

181 AATAGCCATCACCAGCACCATCATTCTGATTCGGAGCTGATAGAAGGTGATCAAAGGGTA

29 P Y S G P L S G P L N K R T T R K S A R

241 CCTTATAGTGGTCCTCTAAGTGGACCTCTAAACAAGAGGACGACAAGAAAGAGTGCCAGG

49 F N I P D S T S S Q D E Q Y L E V T L D

301 TTCAACATTCCTGACTCTACTTCATCCCAAGATGAACAATACTTGGAAGTAACCCTTGAT

69 V R D D S V A V H S V K T A N G A E E D

361 GTTCGAGATGATTCTGTTGCTGTACACAGTGTCAAAACAGCAAATGGTGCTGAAGAAGAT

89 P E L T L L A K G L E K K S N S N I V R

421 CCAGAATTAACACTGCTTGCTAAAGGACTAGAAAAGAAATCTAACTCTAACATTGTCAGG

109 N A S N R F R Q V S Q E I K R L A S F S

481 AACGCCTCTAATAGGTTTAGACAGGTTTCTCAGGAAATTAAGCGGTTAGCTTCGTTTTCG

129 K R P P P G R L D R T K S A A A H A L K

541 AAAAGACCTCCTCCTGGTCGTCTTGACAGGACTAAATCTGCAGCTGCTCATGCTTTGAAA

149 G L K F I S K T D G G A G W A A V E K R

601 GGACTCAAGTTTATTAGCAAGACCGATGGAGGAGCTGGATGGGCTGCTGTGGAGAAACGA

169 F D D I T A S T D G L L P R S R F C E C

661 TTCGATGACATTACTGCTTCTACTGATGGATTGCTTCCTCGTTCAAGATTCTGCGAATGC

189 I G

721 ATAGgtagttccccgtgcaagatcaacttcccttgtatgagttatttaatttttttggtta

191 M K E S K

781 ggactaactgatgctatctgtttgtgaacttgtgaaaataacagGTATGAAGGAGTCAAA

196 D F A G E L F N A L A R K R H I E R D S

841 AGATTTTGCAGGGGAGCTGTTTAATGCACTTGCTAGGAAGAGGCACATAGAAAGGGATTC

216 I G K D E L K E F W E Q I S N Q G F D S

901 AATCGGTAAAGACGAGCTCAAGGAATTCTGGGAACAGATTTCTAACCAGGGCTTTGATTC

236 R L Q T F F D M

961 CAGGCTTCAGACTTTCTTTGACATgtaatgttatattagaaagtgtgaatcatttattat

1021 tatgatttttatttttgtgtgtaacaacaaaaaaacgtgtattctgatttgcattaccaa

244 V D K D A D G

1081 gcgtttaagatgtaacgtaaatgatgaaaattaattgcagGGTTGATAAAGATGCAGATG

251 R I T E E E V K E

1141 GAAGAATTACAGAAGAGGAAGTAAAGGAGgtatagactttatttaatattaaatcaaaca

1201 aatgtatatgatgcacatgatatattccagaagttgactcatatctttctgtagtatcaa

1261 taatttcaatgcatcagagcatgtgcttcatatccgaagaagttttggactaattgagac

260 I I T L S A S A N K L S

1321 aaattttaccattccgatttgcagATTATTACCCTTAGTGCTTCTGCGAATAAACTATCA

272 N I Q K Q A E E Y A A L I M E E L D P E

1381 AATATTCAAAAACAAGCAGAGGAATATGCAGCCTTGATCATGGAAGAGCTGGATCCAGAG

292 N H G Y I M

1441 AATCATGGATATATCATGgtaaataaatgagaaatgctcttcgtgtgaccattctttcgc

1501 ttttttttggtacaatttattgtgataggccattgattgtgtgaatattgtttccaaata

298 I E N L E M L L L Q G P N Q S V R V G

1561 cagATAGAGAACTTGGAAATGCTTCTATTGCAAGGACCAAACCAATCTGTAAGAGTAGGA

317 E S K N L S Q M L S Q K L K P S L D D N

1621 GAGAGCAAAAACCTGAGCCAAATGCTTAGTCAAAAGCTTAAGCCTAGTTTGGATGACAAC

337 P I R R W G R S T K Y F L F D N W K R V

1681 CCAATAAGGAGATGGGGTAGAAGCACCAAATACTTCCTTTTTGATAACTGGAAGAGAGTG

357 W V I A L W I G V M A G L F A Y K Y V Q

1741 TGGGTGATAGCTCTGTGGATTGGTGTTATGGCTGGCCTGTTTGCCTACAAATATGTGCAA

377 Y K R R A A Y E V M G A C V C I A K G G

1801 TATAAACGGAGAGCTGCATATGAAGTAATGGGCGCATGCGTGTGTATTGCAAAGGGAGGT

397 A E T L K L N M A L I L L P V C R N T L

1861 GCTGAGACACTCAAATTGAATATGGCTTTAATTTTACTACCAGTCTGCAGAAACACCCTC

417 T W L R N K T K L G V V V P F D D N L N

1921 ACTTGGCTTAGGAATAAGACCAAGTTAGGTGTTGTGGTTCCTTTTGATGACAATCTTAAT

437 F H K

1981 TTCCACAAGgtattagcttagttaattgccttcacttattcacgccctttctactgaaag

440 V I A V G I T

2041 aaaacagaaaggtaaataaatgggctgcgttctttttccagGTGATAGCAGTGGGAATTA

447 I G V G L H A I S H L A C D F P R L L S

2101 CAATTGGAGTTGGTCTGCATGCAATTTCTCATTTGGCTTGTGACTTCCCTCGCCTTCTTA

467 A S E E K W E L M E P F F G D Q P S S Y

2161 GTGCAAGTGAAGAGAAGTGGGAACTTATGGAGCCATTCTTTGGGGATCAACCTTCAAGTT

487 W H F V K S V E G V T G I I M V V L M A

2221 ACTGGCATTTTGTTAAGTCAGTGGAAGGAGTGACTGGGATCATAATGGTGGTGTTAATGG

507 I A F T L A A P W F R R N K L N L P S F

2281 CAATAGCCTTTACACTGGCTGCCCCTTGGTTTAGGCGTAACAAGCTGAATTTGCCTTCCT

527 L K K L T G F N A F W Y S H H L F I I V

2341 TTCTTAAGAAGCTCACTGGTTTCAATGCCTTTTGGTACTCTCATCATCTCTTCATCATTG

547 Y T L L I V H G Q Y L Y L T H D W Y K K

2401 TCTACACTCTGCTCATTGTTCATGGTCAATACCTGTACCTGACTCATGATTGGTACAAGA

567 T

2461 AAACGgtaagacacttcttaaattgaaatcaacactatttattataaatatttttgaact

568 T W M Y L A V P I I I Y A

2521 gatccttctctgtattggcagACATGGATGTATTTGGCTGTTCCCATTATCATTTATGCT

581 S E R L T R A L R S S I K P V T I K K

2581 AGTGAAAGATTGACAAGGGCACTAAGGTCCAGTATCAAGCCTGTTACCATTAAGAAGgtg

2641 agcagaacacaattagatgttcatttatagtttgtctgtattacattaagaagttatgga

600 V A I Y P

2701 aattagaaaagtggtgcctaaataatcttgtttttgatattgaagGTGGCTATTTATCCT

605 G N V L A L H M S K P Q G F R Y K S G Q

2761 GGAAATGTTCTGGCACTTCACATGTCAAAGCCTCAAGGATTCAGATATAAGAGTGGACAA

625 Y M F V N C A A V S P F E W

2821 TACATGTTTGTCAATTGTGCTGCTGTGTCTCCATTTGAGTGgtaggtgaatttttagttt

2881 agtactaaatgttgcaggtaatattaatactttgctattacttaaaaaagttaaaatttc

639 H P F S I T S A P G D D Y L S V

2941 acatgtcaacagGCACCCATTTTCCATCACTTCAGCTCCTGGAGATGACTATCTAAGCGT

655 H I R T L G D W T R Q L K T V F S Q

3001 TCACATCAGAACTTTGGGCGATTGGACAAGGCAACTTAAAACAGTATTCTCACAGgcatg

3061 tgtttttttatttggaaatgagtggatttataatgcgtaatctgtttccaagattcttat

3121 atactgcccctctgttggcgactcgactactgtggctaattcaatttccctcatgttcct

673 V C Q P P D T G K S G L L R A D

3181 tcgatttcacagGTGTGTCAACCACCAGATACGGGGAAAAGTGGACTTCTCAGAGCGGAT

689 G F Q G N N C P S

3241 GGCTTTCAAGGAAATAATTGCCCAAGgtaagttctttcaacttggggaaaatgcttactc

3301 tattcacatgtattttcatgaaataagtaatacaataaccattttcaaaaggagtgcata

3361 tgtaaagaggggtacattctgttgtgcatacgtgacaagcattatatcaggtcaacttgt

3421 aaattatgcgtgaatctgggggtggttggccacagcaatctcatataaggaaaattcttg

3481 catgcgcaacttagctacttgcccttagggtctcatcctcactgtcatttctttgggcgg

3541 gttagttgggtgggtgggggaggcggattaaaataccacaccagaagacaaaataagagg

3601 attagaatctttgacagaattgtatattatttctattttaactacggtaatatatgttcg

3661 gacacctatctatagtgcccacgcaatcattgtcattccatgccccaccatagacaggga

3721 catctaacaaacacatgcacatggcgctttgtatggaagtggcccttgttcttccctttg

3781 ttttgaaattctgatccattgttttggttgtataaggtaacgctagtgaatttcttgaac

698 F P R V L I D G

3841 tgattacttaattgtggtattgacgatgatgaacagTTTTCCAAGAGTACTGATTGATGG

706 P Y G A P A Q D Y K K Y E V V L L V G L

3901 TCCATATGGAGCACCAGCACAAGACTACAAGAAATATGAGGTGGTTCTGCTTGTGGGTTT

726 G I G A T P M I S I V K D I V N N I R A

3961 AGGAATTGGAGCAACCCCCATGATCAGTATAGTTAAGGACATAGTTAACAATATCAGAGC

746 R E D E E E E N A L E N G T L P K T P S

4021 CAGGGAAGATGAAGAAGAAGAGAATGCATTAGAAAACGGTACATTGCCAAAAACACCTAG

766 P D A Q K R R E N F K T R R A Y F Y W V

4081 TCCTGATGCACAAAAAAGAAGAGAGAACTTTAAAACAAGAAGGGCGTACTTCTATTGGGT

786 T R E Q G S F D W F K G V M N E V A E L

4141 GACAAGGGAGCAAGGATCTTTTGACTGGTTTAAAGGGGTGATGAATGAGGTAGCTGAACT

806 D H N H V I E L H N Y C T S V Y E E G D

4201 GGACCATAACCATGTAATAGAGCTACACAACTACTGCACTAGTGTATATGAAGAAGGGGA

826 A R S A L I T M L Q S L H H A K N G V D

4261 TGCAAGGTCTGCTCTAATTACCATGCTTCAGTCTCTGCATCATGCCAAGAATGGTGTTGA

846 I V S G T R V K S H F A K P N W R S V Y

4321 CATTGTCTCAGGCACTCGTGTTAAGTCTCACTTTGCTAAGCCCAACTGGAGAAGTGTCTA

866 K R T A L N H P N S R V G

4381 CAAGCGCACCGCCCTAAATCACCCTAATTCTCGAGTTGgtaagtccccattcatttttcc

4441 ttttaacaaaacatagtattcttatctttttcacctttctttagaaaaaagcatcttct

4501 gcattattgtgctctttctgtcagttagtaggaaaagtggttaaaaagaacacaaagata

4561 agtatatatattagtggtcgctgggcagtagaggtagtgctttctctgataatatggatg

4621 cttactggtttatctaaaaaattaatcaccattttctgctagggaaagctttctagttaa

879 V F Y C G A P A L T K E

4681 caattgtctcttccctgactgcagGGGTGTTTTACTGTGGGGCACCAGCGCTAACGAAAG

891 L R H L A S D F S H K T N T K F D F H K

4741 AGCTACGCCATCTAGCTTCGGATTTCTCGCATAAGACAAACACCAAATTTGATTTTCATA

911 E N F *

4801 AAGAGAACTTC**TAA**aactttcaatcggtgattaaggaagacagaagaaagaaagaaagag

4861 aggtcttgaattgagttccatcaacatcccactcgttactgaatattctttattggcccc

4921 cttttgttgacatttctattagcctcctttcaataatggcatagtcttattgataaatta

4981 atcagcgagcagaagatagtgcgttggttgattgcaggggcaacccacttaagacagcaa

5041 atgttagtgctcgtctctgtcaatcaggacatatctacaataggatttgtaaagcatttc

5101 cttttgttcatttttctttatttttttctctgctaagaaatatcttgtatgatagaata

5161 gtgaatgtctttcagttcttttgattgattgaaattggacggataaaatataagtattga

5221 gtattaattaagtgtatgtataaactacaagtctttttctaaatctaattatgtagtgaa

5281 tcatcagctaa
