# Supplementary material for: Genomics analysis of genes encoding respiratory burst oxidase homologs (RBOHs) in jatropha and the comparison with castor bean
Source: PeerJ. 2019 Jul 11;7:e7263. doi: 10.7717/peerj.7263 (PMC6626655; doi:10.7717/peerj.7263)
Supplement: File S3 [file peerj-07-7263-s003.pdf]

Supplementary File S3. The gene model for *RcRbohD*. The coding region is marked with uppercase letters, above which is its deduced amino acids. The transcribed untranslated regions, including 5’ UTR, intron and 3’ UTR sequences, are marked with lowercase letters. The start and stop codons are marked with bold letters.

```
  1 cgagctttctctgcattgcatacaatataaagccaggagaaggttctatcgtaccattca
 61 acacacagatacgaaagttgcctaatcaaattcttttattttccatttaaaaaaaaaaaa
121 aaaagtttcttcatcctcgttttctacattcatttttgttgttctccgccctgccagaag
  1              M  R  K  E  T  T  T  T  D  P  H  Q  G  K  G  T
181 gaaaaaaaaaacATGAGGAAAGAAACCACAACTACTGATCCTCATCAGGGCAAAGGCACT
 17 Y  Y  F  S  D  N  T  S  D  T  E  S  I  A  S  D  R  T  A  F
241 TATTATTTTTCAGATAACACCTCGGACACTGAGAGCATTGCCAGCGATCGAACTGCCTTT
 37 S  G  P  L  G  V  G  V  G  A  P  L  H  K  K  R  S  S  K  K
301 AGCGGTCCTCTTGGAGTTGGAGTTGGAGCTCCTCTCCACAAAAAACGTTCCTCAAAGAAG
 57 S  A  R  F  N  L  P  P  E  T  S  S  A  G  V  E  D  P  S  S
361 AGCGCAAGGTTCAATCTTCCACCCGAAACCTCCTCTGCCGGTGTCGAGGATCCATCCAGC
 77 S  Y  V  E  I  T  L  D  I  R  D  D  S  V  A  V  H  S  V  Q
421 TCCTATGTAGAGATTACTCTTGACATTCGCGACGACTCTGTGGCGGTTCACAGCGTCCAG
 97 G  A  N  Y  E  D  P  D  L  A  L  L  A  K  R  T  L  E  N  K
481 GGGGCTAATTATGAGGACCCAGATTTGGCGTTGCTTGCAAAGAGGACTCTCGAGAACAAG
117 S  T  S  L  F  R  N  T  S  S  H  I  K  Q  E  L  K  R  F  A
541 TCTACTTCCCTGTTTCGAAACACCTCTTCCCATATAAAGCAGGAGCTCAAACGTTTTGCT
137 S  L  S  R  R  P  S  A  A  K  R  F  D  R  T  K  S  A  A  A
601 TCCCTGTCAAGGAGACCTTCTGCCGCTAAAAGGTTTGATAGAACAAAGTCAGCTGCTGCT
157 H  A  L  K  G  L  K  F  I  A  A  K  T  G  S  S  A  N  G  W
661 CATGCCCTGAAGGGCCTCAAGTTCATCGCCGCAAAAACTGGATCTTCTGCAAATGGATGG
177 S  S  V  E  K  R  F  Q  Q  L  T  V  N  T  D  G  L  L  H  C
721 TCTTCTGTTGAAAAGAGATTTCAACAACTTACTGTTAATACTGATGGTCTCCTTCACTGT
```

197 S L F G E C I G

781 TCCTTGTTCGGTGAATGCATAGgtactgtttttcattataactaattacatatatattt

205 M N K D S K

841 cctaaattcaatattaaaatttattaacttggattggacagGCATGAACAAGGATTCAAA

211 E F A G E L F R A L A R K H N I N G D S

901 GGAATTTGCTGGTGAATTATTTCGCGCGCTTGCTCGGAAGCATAACATAAATGGTGATTC

231 I N K A Q L K D F W D Q I S D E S F D S

961 TATTAACAAGGCACAGCTAAAAGATTTCTGGGACCAGATCTCTGATGAAAGCTTCGATTC

251 R L Q I F F D M

1021 CCGGCTCCAAATATTCTTTGACATgtaagaattacccttcaaattttaattaaaccattc

1081 aacgaattagtcctcataatatttatatatgtatataattagtacctgaaaatgcgaatg

259 V D R D A D G R I T E E E V T E

1141 catgcagGGTGGACAGAGACGCTGACGGAAGAATCACAGAAGAAGAAGTAACAGAGgtaa

1201 agcgtacagtgcgatacctggtcaacgttgacttttccaaacctaacctaaataattaat

1261 ggacaattccatagactactactacttattaattactagtttagtttaaactaacgacat

275 I I S L S A S A N K L S N I Q K

1321 gatcaatttgtagATTATCAGTTTAAGTGCTTCTGCAAACAAGCTCTCCAACATTCAGAA

291 Q A K E Y A A L I M E E L D P D N A G Y

1381 ACAAGCCAAGGAATACGCTGCCCTAATTATGGAAGAATTAGACCCAGATAATGCTGGATA

311 I M

1441 CATCATGgtacctacctattattattattattgtaactgcattattaattgctatctttt

313 I Y N

1501 aatttaatttgtctttcccgcccttacttaactccaatttttcttaatacagATTTATAA

316 L E T L L L Q A P N Q S V R I G D S R V

1561 TCTAGAAACACTACTACTGCAAGCTCCAAACCAATCTGTCAGAATTGGAGATAGCCGAGT

336 L S Q L L S Q K L K P T Q E T N P L K K

1621 TTTGAGTCAACTACTGAGTCAGAAACTCAAGCCTACACAAGAGACCAATCCATTAAAAAA

356 W Y Q K A K Y F F L D N W Q R V W I M A

1681 ATGGTATCAAAAGGCCAAGTACTTTTTCTTAGATAACTGGCAGAGAGTGTGGATCATGGC

376 L W L G I V A G L F S Y K F V Q Y R N K

1741 ATTGTGGCTGGGAATCGTAGCTGGTTTATTTAGTTACAAGTTTGTACAGTACCGAAATAA

396 A V F E V M G Y C V C I A K G G A E T L

1801 GGCTGTATTCGAGGTAATGGGCTACTGTGTTTGCATTGCCAAAGGAGGGGCAGAGACACT

416 K F N M A L I L L P V C R N T I T W L R

1861 TAAATTCAATATGGCCTTGATTTTACTGCCTGTCTGTCGGAACACCATTACTTGGCTCAG

436 N K T K L G V A V P F D D N L N F H K

1921 AAACAAGACTAAGTTGGGAGTGGCTGTGCCTTTCGACGACAATCTCAATTTCCACAAGgt

1981 aatcatatagtaggaaattttaattagctagagcctagagcatgggggaattcctaattc

455 V I A V G I A I G I G L H

2041 tcttttgggttgatcgatcagGTGATTGCCGTTGGAATTGCCATTGGGATTGGGCTACAT

468 A G A H L T C D F P R L L H A T E E E Y

2101 GCAGGTGCGCATTTAACTTGTGATTTTCCACGGCTTCTCCATGCCACTGAAGAGGAATAC

488 E P M E P Y F G E D Q P E N Y W W F V K

2161 GAGCCTATGGAGCCTTATTTTGGGGAAGACCAACCCGAAAACTACTGGTGGTTTGTAAAA

508 G V E G V T G I I M V V L M A I A F T L

2221 GGGGTGGAGGGGGTCACAGGAATTATAATGGTAGTCCTAATGGCTATAGCCTTCACACTA

528 A T P W F R R N K L N L P K P L K K L T

2281 GCCACTCCTTGGTTTAGGCGTAACAAGCTCAATCTTCCCAAACCCCTCAAGAAACTAACA

548 G F N A F W Y S H H L F V I V Y A L L I

2341 GGTTTCAATGCCTTCTGGTATTCTCATCATCTCTTCGTAATTGTCTACGCTCTCCTCATT

568 V H G I Y L Y L T K T W Y H K T

2401 GTCCATGGTATTTACCTCTACCTCACCAAGACATGGTATCATAAAACGgtcagtcttcaa

2461 actctcttcttcttaatgattttgagttttctccactcgcgaaaatctgatttccatatt

584 T W M Y L A V P I I L Y A C E R

2521 ctctactatacagACATGGATGTATCTAGCAGTGCCCATAATCCTTTATGCATGTGAGAG

600 L I R A F R S R I K S V K I L K

2581 GTTGATCAGGGCATTTAGATCACGAATCAAGTCCGTTAAGATTCTCAAGgtattaacgga

2641 aagctcttgcccattttagaatatttgttattaatattgtattgcagataattgtaaatc

2701 tttaatttagtgctagccagtcactgttttccgtattcttaatgcatgcctactgaaatg

616 V A V Y P G N V L A L H M

2761 atgaacacgactcatatatagGTTGCTGTATATCCGGGAAATGTCCTAGCATTGCACATG

629 S K P Q G F K Y K S G Q Y M F V N C A A

2821 TCAAAGCCACAAGGCTTCAAATATAAGAGTGGACAGTACATGTTTGTCAACTGCGCTGCA

649 V S P F E W

2881 GTTTCTCCCTTCGAATGgtaactacctctaccttattatccgaactgtcattttttggtta

655 H P F S I T S

2941 gccattgaggaataatataattgaatatgtatatttgcagGCATCCATTTTCTATCACAT

662 A P G D D Y L S V H I R T L G D W T R Q

3001 CAGCACCAGGAGATGACTACCTCAGTGTGCATATTCGAACTTTGGGTGACTGGACCCGCC

682 L K T V F S E

3061 AGCTCAAAACTGTTTTTTCTGAGgtctgttataattagttcacaggctactgtataatct

3121 gttctttttctctttttttggtctcttcaatggaccgcctaattaatctcacctgcactat

689 V C K P P T A G K S G L L

3181 cgttttcattggttattaatagGTTTGTAAGCCTCCAACTGCTGGTAAGAGTGGACTGCT

702 R A E G N N P G

3241 CAGAGCTGAGGGAAACAATCCTGGgtaattgcgattatatagtatataataataacaacg

3301 ttagttagtcggaagttttaaatgtagtgttttatttgggagcagcaaaatctaatagtg

710 F P K I L I D G P Y G A P A Q D

3361 gaaaggaacagGTTTCCAAAGATATTGATAGACGGACCGTACGGAGCTCCAGCACAGGAC

726 Y K K Y D V V L L V G L G I G A T P M I

3421 TACAAAAAATACGACGTGGTGCTGTTGGTAGGGCTAGGGATAGGGGCTACACCCATGATT

746 S I V K D I I N N M E E D D G E L E S G

3481 AGCATTGTGAAGGACATCATTAATAACATGGAGGAAGATGATGGTGAGTTAGAGAGTGGG

766 R A T A A N N N N S S N N R T R K A Y F

3541 AGAGCGACAGCTGCAAATAACAACAATAGCAGTAACAACAGGACGAGGAAAGCCTACTTC

786 Y W V T R E Q G S F E W F K G I M N E V

3601 TATTGGGTGACGAGGGAACAAGGTTCATTCGAGTGGTTCAAAGGGATAATGAACGAGGTT

806 A E M D D K G V I E L H N Y C T S V Y E

3661 GCAGAAATGGATGATAAGGGGGTGATAGAGCTGCACAACTACTGCACCAGCGTGTATGAA

826 E G D A R S A L I A M L Q S L H H A K N

3721 GAAGGAGACGCCAGGTCAGCCCTGATTGCAATGCTTCAGTCGCTCCACCATGCCAAAAAC

846 G V D V V S G T R V K S H F A K P N W R

3781 GGTGTCGATGTTGTTTCGGGGACAAGGGTCAAATCCCACTTTGCCAAGCCGAATTGGCGG

866 Q V Y K K I A L H H P D A R I G

3841 CAAGTCTACAAGAAAATTGCACTCCACCATCCTGATGCAAGAATTGgttggttcacctaa

3901 cctatcccattttatttcttctattgactttttatggaatactgattatataaagaactg

882 V F Y C G A P A L T K E L R Q L A

3961 aatgcagGAGTTTTCTACTGTGGGGCACCAGCACTAACAAAAGAGCTAAGACAACTAGCT

899 L D F S H K T S T K F D F H K E N F *

4021 TTGGACTTCTCCCACAAGACCTCCACTAAGTTCGATTTTCACAAGGAGAACTTT**TAA**att

4081 tgagcaacagggtatacagatacatacgattctgtggtagcaaattcatatattatggct

4141 gactcactggtatacaacgtcatccaattagctacaccgggaatagttattatacgtagt

4201 tcctctaatttaatttattcgagtttaaatttcaattttaagaacagagaaattcttgat

4261 acaaaaaaagaaaaagaaagaagatgtaattatatttgatggagctgctaatcaagaatt

4321 aaagttgatggaatttgataagcctgtgtttgttccattgattgcgtttagtgggggatt

4381 taccagaagaaactatgggaactaaagtcacttttcttacatatatttaatctatg
