# Supplementary material for: Genomics analysis of genes encoding respiratory burst oxidase homologs (RBOHs) in jatropha and the comparison with castor bean
Source: PeerJ. 2019 Jul 11;7:e7263. doi: 10.7717/peerj.7263 (PMC6626655; doi:10.7717/peerj.7263)
Supplement: File S4 [file peerj-07-7263-s004.pdf]

Supplementary File S4. The gene model for *RcRbohN*. The coding region is marked with uppercase letters, above which is its deduced amino acids. The transcribed untranslated regions, including 5’ UTR, intron and 3’ UTR sequences, are marked with lowercase letters. The start and stop codons are marked with bold letters.

1 gcaccactaatctcctctaacactttaggcttaatcccatagcaccactaatctcttcta

61 cgattccacaaacaccaagccataacagcaaagatgacatcccactacatatcacaacac

121 cacaaactgctgccaccgctcacagtcggagcaacaatattgatatcacccaaaatctag

181 ccattcaagctcgcccacatccagctgtttcaagatcgccatatcttttgcccatcgaga

241 tgaatcagtcattactagttccagcctctaccatcgccaaaatctctacaacaaccatag

1 M A A V E A A G R R C R L R F V D

301 acgataatg**ATG**GCGGCGGTTGAGGCTGCAGGGCGACGGTGCAGACTGAGATTTGTTGAC

18 I E W N D A E R R F D R L A S A G K G P

361 ATTGAGTGGAATGATGCGGAAAGGCGTTTCGACCGGCTAGCTTCCGCCGGAAAAGGCCCT

38 E A V V K W S E F G C C L G

421 GAAGCGGTGGTGAAATGGTCTGAATTTGGTTGTTGTTTAGgtataataagagagttttgt

481 attctcatactttgttccttggatttcctattcttctttctttttgcttacgtttttct

541 taacagatttagagagaaattaagggaacatatagccgatagagttgcatgagtttttgt

601 ccatgttatgtacttggcactagcctatgctctctcttttagtcatgtcacaatctcctg

661 tttcagaggagataatttaaaaaagaaagaaaagaaaaaaaaacttactagacaagtgat

721 cattgcagggaagagccaatgacatcaacaaaagaaaaaaaaaaaggctaggaatttctg

781 ttttctaatttttaaaatggacaattctcgcatattttaatatgtttattcaatatactt

841 aattatttaaatctaataaatataaaatataattctaataaatgggcagttttcttttca

52 I E Q S P E F A K E I L M A L R G

901 tatttacagGCATCGAACAATCGCCAGAATTTGCCAAAGAGATTCTAATGGCACTAAGAG

69 R R R D C R S D I T K R E L Q I Y W Q R

961 GAAGAAGAAGAGATTGCAGGAGTGATATCACAAAAAGAGAACTCCAGATTTACTGGCAAC

89 L T D P C F D S R V K I Y F D M

1021 GATTGACTGACCCTTGCTTCGATTCAAGAGTGAAAATATATTTTGACATgtaaggatcat

1081 gcagatatgataattgtttgtgaaattatttcttatatgtatgtattgtttaattgatga

105 C D R D M D G K I N

1141 ggatgttttatttgtggccttattttcagGTGCGACAGAGACATGGATGGAAAGATTAAT

115 E I D I K Q T I L Q S C S T N K V S L T

1201 GAGATTGACATTAAGCAGACTATTCTGCAAAGTTGTTCAACAAATAAAGTGTCTCTAACC

135 H E Q A Q E Y A A L A I E A L D T Q H S

1261 CACGAGCAAGCACAGGAATATGCTGCCTTAGCCATTGAAGCTCTTGACACTCAACACTCG

155 G F I Q

1321 GGCTTCATTCAGgttcactgtttgaccccttgcttccttctacagtttggtccacagcat

1381 tattatacatccggttacttttacttattaaaaagtaaattaattaattaatacatctat

1441 tttttaaaaaaaatctagcagtcattcctttatgaggttattactatttgagcaaaatt

1501 aaataggcttttcttaaaatttttttggaccattttacttcaaaaaaatttatcattttt

1561 aattcagtttaactctttttagaagagtgaattacattgtttatattaaaagaataaatt

1621 taataaaaaataattaaaatcaatttttagaattgattttaataaataagtaaatcatct

1681 cgttggtgtgaaaatttttttgccacttatatccaaatgataccatttttttaatttatttt

1741 ttttagtatcatatacctattatttttatttcaattaatggtagctaatagagacttgcc

1801 taaaattcatgtgagttaagaactcgccaatattcaacaatccacttatactactaacat

1861 atcaataggcgaaccaacattccacgtagctataaagtattactcctgaaaaattgaaat

1921 ttctgatctctaggaccacactaaatatagaaataactagcacttctcttataaatacct

1981 atttaaaatatcaataaaaaagtaatgcttatacaacctttaaacaatattccttaaact

2041 cctactcaaaacaaatcatattgttgttatcttacactcttagtaacatcattatccacc

2101 ggaattcgatttttttaacatgaatcctagtaggtggcatcaattgagacaaatatagta

2161 gttgtggtactataaagaaacgaatcgaagaaaacgatatcatttagttattaagtggca

2221 aagctttagtactaatatgacaatttatttaaagtcaagtgtgatatggatggtaaatag

2281 attactaacaaggagctctacaaactaattaaaattaaaattttaaaaaattaaaatttt

2341 aatgctaaaaagaaataaattaaagtattagtactaaaattttagtaccaacatagctat

2401 ttagcctattttagtccctaaagtttattaaactctacaaactaattttgtcagtaattt

159 P D Q L I T L F R Q S I P T G S L A

2461 gtatgcagCCAGACCAACTAATAACTCTCTTCAGACAAAGCATACCAACAGGCTCATTAG

177 M A Y K N K N N L N N Q E Q P T T S K T

2521 CAATGGCGTATAAGAACAAAAACAATCTAAATAACCAAGAACAACCAACTACGTCGAAAA

197 A A E I M F R T H W R Q G W I T L F W L

2581 CTGCAGCAGAAATAATGTTCCGAACACATTGGAGACAAGGATGGATAACGTTGTTCTGGT

217 V V C F S L F M W K F I Q Y R H R T A F

2641 TGGTAGTTTGCTTTTCACTTTTCATGTGGAAGTTCATTCAATACAGACACAGAACAGCAT

237 Q V M G Y C L C T A K G A A E T L K F N

2701 TTCAAGTAATGGGTTATTGCCTTTGCACCGCCAAAGGAGCAGCTGAAACCTTAAAATTCA

257 M A L I L L P V C R N T I T W L R R K S

2761 ACATGGCTTTGATCCTTCTACCTGTTTGTCGGAATACTATTACATGGCTTCGGAGGAAGT

277 G F S S F V P F N D N I N F H K

2821 CAGGTTTCAGTTCTTTTGTACCGTTCAATGATAACATCAACTTCCACAAGgtatttttt

2881 tatttttattttatctacatatgttctctctttatttcactcttgtttattgcttaatta

2941 tcagtgctatcaatttatgttagtgggattaattcaaattagaattgattgttgtagtaa

3001 ttagtaatttctttatttggtggattatgttgtaatagttatgataaatttagatttcgt

3061 tgtgtctatttgttcaagttattgaacagattgatacattgtaacagtgctctcacattt

3121 tgttatagaactgaaatatatgtcactcaggttctgttagaataatttttctagtgcaat

3181 gtcaaacgaataatagctcttgaaaccttcacaaacattaactaacttctgcaattttca

293 L I A G G I V V G V I V

3241 gtgacaaaagtgtttctttttacagCTAATTGCAGGAGGCATAGTGGTGGGTGTGATAGT

305 H G G T H L A C D F P R I S G S D R S I

3301 CCATGGGGGAACTCACCTTGCTTGTGATTTCCCAAGAATTAGTGGGTCGGACCGATCAAT

325 F R Q T I A G R F G Y H Q P S Y I Q I L

3361 TTTTCGGCAAACGATAGCCGGCCGATTTGGGTATCATCAGCCATCATATATCCAAATATT

345 A T T E A A T G I A M V I L M A I A F L

3421 AGCAACGACAGAAGCTGCAACTGGAATTGCCATGGTAATACTAATGGCAATTGCATTTTT

365 L A T K W P R R Q S A S L P K S I R N V

3481 GCTTGCAACAAAATGGCCTCGTCGGCAATCAGCTTCTCTGCCTAAATCCATTAGAAATGT

385 T G Y N T F W Y S H H L L I L V Y V L L

3541 GACTGGATATAATACTTTCTGGTACTCACACCACTTGCTCATTCTTGTCTATGTGTTGCT

405 I I H S M F L F L T D N V T E K T

3601 CATTATTCACTCCATGTTCCTCTTCCTTACTGACAATGTCACTGAGAAAACGgtatgtat

3661 ttcagtttggtttttcagttaaacccatatttagatgttaagttgagtcacttcacacac

3721 tactgcaattacttaagtcctgtggtttatgtacccataagctaagtctggcagttggtg

3781 catatgtcaaattgaaaggactcaagttcgagtttttctctcttatttgcaattaaaaaa

422 T

3841 tagaaaagccatgcggttgatgcattgatagagattaaaattgagacattttggtgcagA

423 W M Y I A L P V M L Y A G E R V A R A V

3901 CCTGGATGTACATTGCCTTGCCAGTTATGTTATATGCCGGAGAACGTGTTGCTCGAGCTG

443 R S G F Y D A K I L K

3961 TAAGATCAGGATTTTATGATGCAAAAATTCTGAAGgtaattgataaatgcaacttactag

454 A

4021 attataaaattaataatgttaacattatttagctaaggacatgttactgtattagtagGC

455 S I Y P G K V L S L K L H K P Q G F K H

4081 TAGTATTTATCCAGGAAAGGTTTTATCTCTGAAGTTGCACAAACCACAAGGTTTCAAGCA

475 K S G M Y V F L Q C P Q I S P F Q W

4141 CAAGAGTGGGATGTATGTCTTCCTACAGTGCCCTCAGATTTCTCCATTTCAATGgtaact

4201 atacaacatttctcgttatttttctttgtatcatttccttcttttatgtactgcctcaga

4261 gaatccacaatttgtaatggtaaaatttgaacccctgatcaattgcaacaaggaaagcac

4321 tctactaattgactactagtccaaataatcaaattgtagcaagttaaccaaacaccttgt

4381 ctttcttttgcttgtacagcaaatatgtttagcccatataccatctaataagtcaaaata

4441 ttcctctaattttttccctcttgcatcaagtaagcttttatctaggtatatgcccgtctc

493 H P F S L T S G P E D D H L S V

4501 tcatttttcagGCACCCATTTTCATTAACTTCAGGGCCAGAGGATGATCACTTAAGTGTG

509 H I R T L G D W S Y K I Y Y L F Q E

4561 CATATTAGAACTCTTGGAGATTGGAGTTACAAAATATACTATCTTTTTCAAGAGgtaaaa

4621 gaactaactgtaataaaacacacacatacttgattgcctatcaacttgaagaatgagacc

527 A A L S G G K E Y P K

4681 tatttcaagattcttctggggttttgcagGCAGCACTATCAGGGGGCAAGGAATATCCAA

538 I Y I D G P Y G A S S Q D H V K Y D I V

4741 AAATATACATTGATGGACCCTATGGTGCTTCTTCCCAAGATCATGTCAAGTATGATATAG

558 M L I G L G I G A T P F I S I L K D I A

4801 TCATGCTAATTGGGCTTGGCATTGGAGCCACACCTTTCATTAGCATTCTTAAAGACATCG

578 N D L Q M

4861 CAAATGATCTCCAAATGgcaagtaatcaatggcttatgctctactctccatatatataat

4921 agagataaatataaactatttgtggttttgcagatttataactctgcgcgtgtcttttc

4981 tgttaacaatttaaaatatatttactctatcagtattttcagatatattcttgttacatc

5041 attagtaattggtgacttattgacatgtatcattcttcggctaatatggcactgagtaat

5101 atatatataaaaaaaagaagccaaaatgagtaaaatactatcttaaattatcacatatga

5161 aaaaggaaaatgctaaaaaactaaattatcttactttttcctatactgtttcacttattc

5221 aatatctgttgttttctctcaactatcaatctaaatatgtgttgaaaaaagttgctatag

583 A N C E E C N I S R G P L K A Y F Y W V

5281 GCCAATTGTGAGGAATGCAACATTTCAAGAGGTCCTTTAAAAGCTTACTTTTATTGGGTT

603 T R E Q S S F D W F R D V L W E T S K I

5341 ACAAGAGAACAAAGCTCTTTCGACTGGTTCAGGGATGTCCTGTGGGAAACATCCAAAATT

623 N Q K K

5401 AACCAAAAGAAGgtaacttaacattactaattcccttgcttttgttctctccctatggct

627 S V V E M Q

5461 tatggtacatctacatcttatttcctctggtgatccttgcagTCTGTCGTAGAGATGCAA

633 Y F L T N V Y Q E G D A R S A I I S A I

5521 TACTTCTTAACCAATGTTTATCAAGAAGGGGATGCAAGATCTGCTATAATAAGTGCCATA

653 Q A L H L A K K G I D I I S W S Q

5581 CAAGCACTGCATCTTGCTAAGAAAGGCATTGACATCATATCTTGGAGTCAGgtaaaaata

5641 tattactaaatgttgttttttttcccacttctgtaaatatgctaaattgcttatacttggt

5701 ttccttttacgagggagctttgagcctgacgcctctaactgaatatctaaatgatggagc

670 V R T Q F G R P N W F S I F S K M S S

5761 tgtagGTACGCACGCAATTTGGCCGTCCGAATTGGTTCAGCATTTTCTCAAAAATGTCAA

689 K H R G E R I G

5821 GTAAACACAGAGGAGAACGAATAGgtaaccattttatgtacctttcataatttcatttag

5881 tatatttctatttttctccattacagccaaactatatggcttaagatgtgaaaccaatat

5941 tcaaaagctagtaagaaaaaaaaacaatagtgagctttgagatttagtacggtgtatgat

6001 atttattattattttgattataaaaaattaaatttatagatacaatttaattatttttc

6061 atattattaatttataatattttaaaaaataatagcaaattaactatttttagatgtcct

6121 tgatttcttcttaaaactttattagtgcaataatataaaaattagaattataaattatta

6181 tacaannnnnnnnnnnnnnnnnnnnnnnnnnnnnnnnnnnnnnnnnnnnnnnnnnnnnnn

6241 nnnnnnnnnnnnnnnnnnnnnnnnnnnnnnnnnnnnnnnnnnnnnnnnnnnnnnnnnnnn

6301 nnnnnnnnnnnnnnnnnnnnnnnnnnnnnnnnnnnnnnnnnatatgctattttataaaa

6361 taattattatctaattagaaaactaatttattattaatatgttatttttttaaaattagaa

6421 aagtatttaattacaaatgaaacttattaattaataaattaatagaaaaaaaccaatgac

6481 tcttcataatcaataaattaattgtgggtgttcttatatatttattttcgaagtttttttg

697 V F Y C G P S A L G N E L E R L C T

6541 acacagGGGTCTTCTACTGCGGTCCATCAGCACTGGGAAATGAACTGGAGAGATTGTGCA

715 K F S T K T E T R F V F H K E N Y *

6601 CCAAATTCTCAACCAAAACAGAAACTAGATTTGTATTTCACAAGGAGAACTAC**TAG**tgat

6661 gtcctgttgtatagattcccaaatggggaatctatagagcaattgcctaatatagtaaca

6721 gaataaccgatagagattcatatttggacgctaagactcttgagatgatatatgtttttt

6781 tctttcccttttcaacggaaaaaagatagatgtaattgataaatcaaaagtcatacaacc

6841 ataattctcctgagccagctagacgggctgacaagagaatgagttggggctttcttttgg

6901 ctacatcgtgagccacccagttaacattacgtgggtacaaaactaaacaaaccgctagca

6961 aagttctcgctaagattggtgatgtcttccattttaactgcaatttcctgtggaggagag

7021 gttacaccattgacctgttcttctgtaccactagtcggttgtcttctattacgtctcggt

7081 cggtgccataggagtattatctattctcatctataaatttttatataaacaatctatctc

7141 tgcttcttatcatttttagaaaatatgattaattctaaattatatttccagcccaataa

7201 gcaacgatacatgacaaacttatcatgtttaattattatacatattttaaaagtaaaaaa

7261 tacgtcaactctattattacattctttaaaactatttcctagcacgtgatattagtaaga

7321 tttggtttagtaattaaaaggttatggcatttgatttaaaaaaaaaaacatttcaatgaa

7381 attttttaaatatcatttgatactattggtagttaaaatgatttttttaaaaaactgatac

7441 aacataaaaattaatattaaactaaaataaaaaaaaatgcatttaaaatgaaatatatttt

7501 tttatgtttctaaatatatcatttttattttgatttaaaatttaatttatttaagtcaa

7561 aaaatttaacataatataaacatcacatataaataaaaatacatatcaaaattacaataa

7621 tatatgaattaaaattaaataacaaaaaaacagaaacatataaacatcaaaaaataatat

7681 acatatatacaaatacaatatacataaaatagacatataaatttatcataaaaaattttc

7741 agcgtatgcatcaagataagtttacatggtattaagcaaaaaataattaatatatagttt

7801 attctagtttaaattctttatttatttaactaaaaaaattcaaaataatataaacatcac

7861 ataacaaatacaacac
