# Supplementary figures and images for: Genomics analysis of genes encoding respiratory burst oxidase homologs (RBOHs) in jatropha and the comparison with castor bean
Source: PeerJ. 2019 Jul 11;7:e7263. doi: 10.7717/peerj.7263 (PMC6626655; doi:10.7717/peerj.7263)

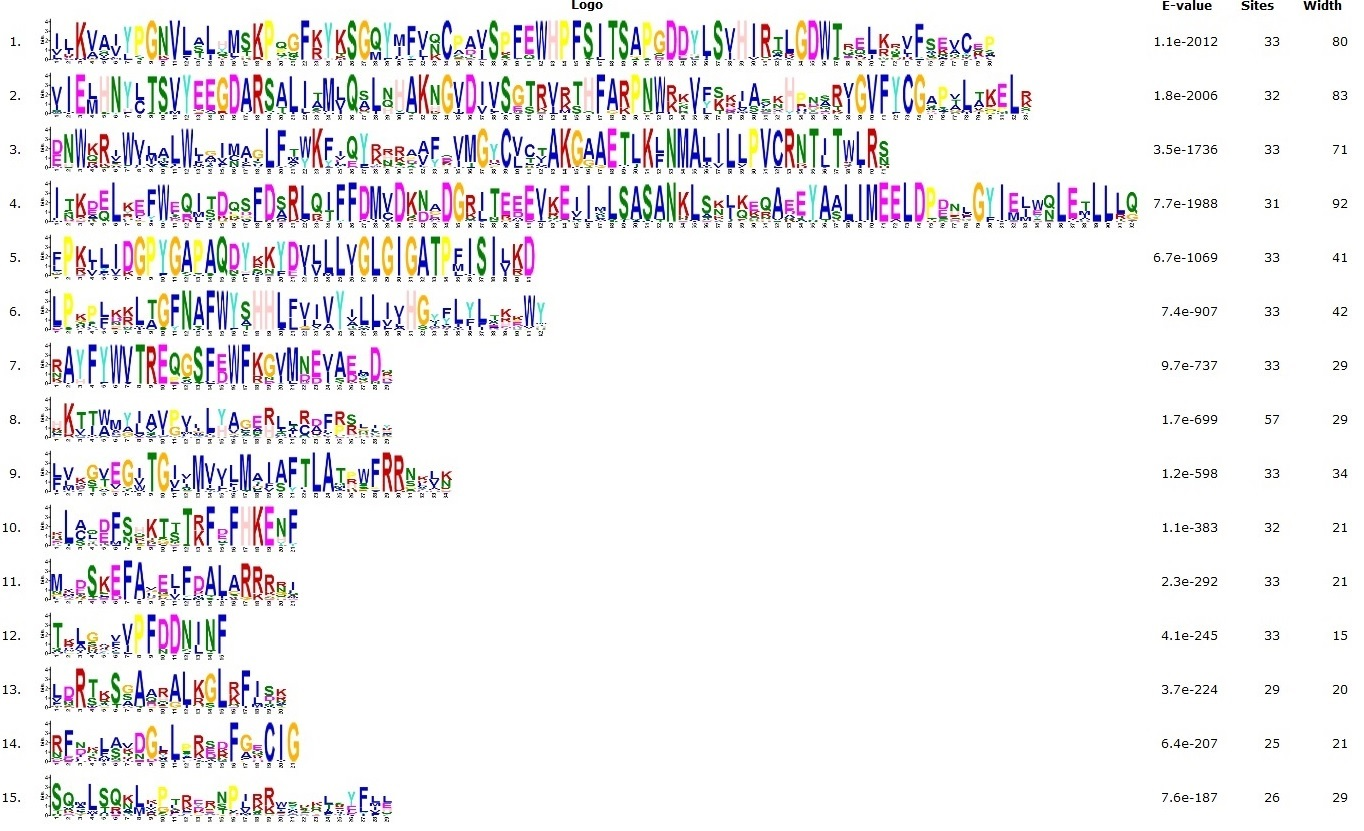

Supplement: Fig. S1 [file peerj-07-7263-s005.png]

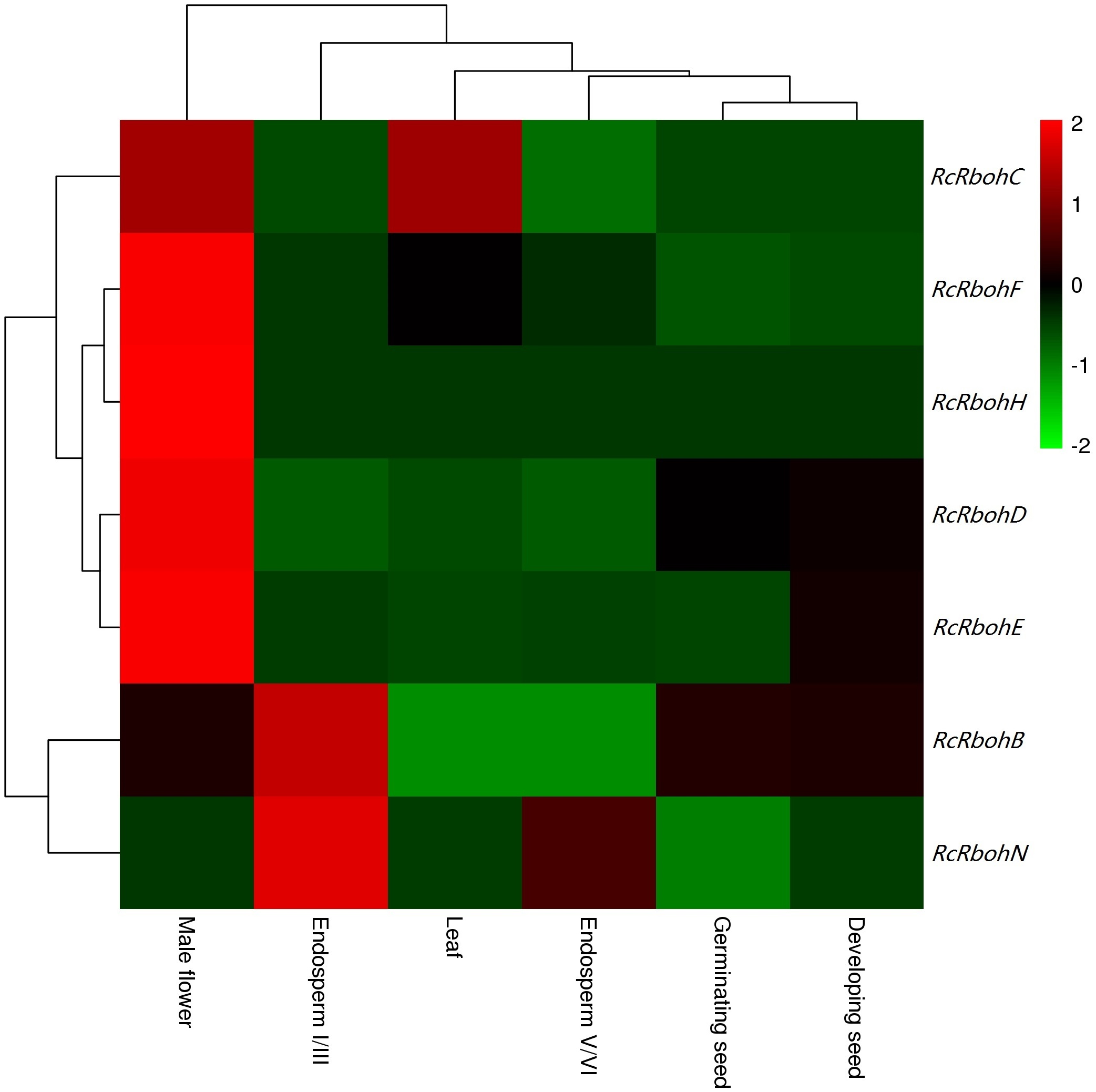

Supplement: Fig S2 — Color scale represents FPKM normalized log_10 transformed counts where green indicates low expression and red indicates high expression. [file peerj-07-7263-s006.png]
